# A Morphological and Immunohistochemical Study of the Tumoral and Inflammatory Cells in Pancreatic Ductal Adenocarcinoma

**DOI:** 10.1155/2020/6148286

**Published:** 2020-09-29

**Authors:** Vlad Herlea, Elena Stoica Mustafa, Andreea Cristina Iorgescu, Nicolae Catalin Pechianu, Dragos Cretoiu, Maria Sajin, Simona Olimpia Dima, Catalin Vasilescu, Cezar Stroescu, Constantin Ungureanu, Traian Dumitrascu, Vladislav Brasoveanu, Minerva Ghinescu, Irinel Popescu

**Affiliations:** ^1^Department of Pathology, Fundeni Clinical Institute, Bucharest 022328, Romania; ^2^“TituMaiorescu” University, Faculty of Medicine, Bucharest 031593, Romania; ^3^Center of Excellence for Translational Medicine, Fundeni Clinical Institute, Bucharest, Romania; ^4^“Carol Davila” University of Medicine and Pharmacy, Bucharest 020021, Romania; ^5^Center of General Surgery and Liver Transplantation, Fundeni Clinical Institute, Bucharest 022328, Romania

## Abstract

This study is aimed at investigating tumoral and inflammatory cells and the significance of the prognostic factors of pancreatic ductal adenocarcinoma (PDAC); it is also aimed at determining the role of immunohistochemistry in the diagnosis and prognosis of this neoplasm. *Materials and Methods*. 230 cases of pancreatic ductal adenocarcinoma were included in the study group; these cases were selected from the archives of the Department of Pathology of the Fundeni Clinical Institute over a ten-year period. Immunohistochemistry was performed using the following antibodies: MUC 1, CD 34, Factor VIII, CD 68, MMP-7, CEA, p21, p53, and Ki 67. *Results*. There were 133 male (57.8%) and 97 female (42.2%) patients included in this study, with ages between 20 and 81 years old (mean age: 58.2 years) and with tumors located in the pancreatic head (*n* = 196; 85.2%), pancreatic body (*n* = 12; 5.2%), and pancreatic tail (*n* = 20, 8.7%), as well as panpancreatic tumors (*n* = 2; 0.9%). Patients presented with early stages (IA and IB), with low pathologic grade (G1), with small size tumors (less than 1-1.5 cm), with tumors located in the head of the pancreas, (p53: negative; p21: positive; and CD 68: positive in peritumoral tissue), with low nuclear index (Ki 67 < 10%), without metastases at the time of surgery (had a better prognosis), and with a survival rate of about 7 months. *Conclusions*. Immunohistochemistry is useful for an accurate diagnosis, differential diagnosis, and establishment of additional factors that might have a prognostic importance. It is recommended to study peritumoral tissue from the quantitative and qualitative points of view to increase the number of prognostic factors. This study represents a multidisciplinary approach, and it is a result of teamwork; it presents histopathological methods of examination of this severe illness and describes only a part of the scientific effort to determine the main pathological mechanisms of this neoplasm.

## 1. Introduction

Pancreatic cancer is an aggressive neoplasm with a poor prognosis. It is the fifth leading cause of death in Europe [1] and the fourth most common cause of cancer-related deaths in the United States [2], mostly because it is often discovered at an advanced stage [2–5]. The response to oncological therapy is limited [6], surgery being the only effective method of treatment for resectable tumors. [2, 7]

Most of these neoplasms are represented by pancreatic ductal adenocarcinomas (85%) [1]. It is rarely diagnosed in young people, and most of the patients are over 60 years old [8]. It grows and spreads very quickly.

Diabetes and chronic pancreatitis are two of the most important risk factors, but prognostic factors are still debatable and challenging [9–12].

This study is aimed at investigating tumoral and inflammatory cells and the significance of prognostic factors of pancreatic ductal adenocarcinoma (PDAC); it is also aimed at determining the role of immunohistochemistry in the diagnosis and prognosis of this neoplasm.

## 2. Materials and Methods

230 cases of pancreatic ductal adenocarcinoma were included in the study group; these cases were selected from the archives of the Department of Pathology of the Fundeni Clinical Institute over a 10-year period. The selection was made from radical surgical resected specimens. Duodenal, common bile duct, and ampullary neoplasms were excluded.

All cases were staged according to international criteria [13, 14]. From the surgical specimens, tumoral and nontumoral tissue, resection margins, and lymph nodes were carefully sampled. Peritumoral inflammatory infiltrate was studied using a pancreatic tissue sample, taken 1 cm from tumoral/peritumoral junction.

Tissue samples were fixed in buffered formalin (10%) for 10-20 hours and embedded in paraffin (57-58°C). From every paraffin block, serial sections were cut into 3-4 *μ*m slices and then routinely stained with hematoxylin and eosin for histopathological diagnosis.

### 2.1. Morphological Analysis

The studied cases were distributed into the following 7 age groups: 20-29 years old, 30-39 years old, 40-49 years old, 50-59 years old, 60-69 years old, 70-79years old, and over 80 years old. Age distribution was correlated with gender, size, and site of tumor. Tumor size was assessed in cm, and the cases were distributed into 5 groups: 0.1-1.0 cm, 1.1-2.0 cm, 2.1-3.0 cm, 3.1-5.0 cm, and over 5 cm. The tumor site was distributed according to anatomical regions in the head, body, tail and, in some cases where the entire organ was affected, panpancreatic.

Histological characteristics assessed for each case were tumor grades G1, G2, and G3 (according to the guideline criteria in use [12, 13]); the presence or absence of lymph node metastases (N0/N1); the presence or absence of vascular invasion (V+/V-); the presence or absence of perineural invasion (Pn+/Pn-); and the presence or absence of endoluminal necrosis (Ne+/Ne-).

### 2.2. Immunohistochemistry

For immunohistochemistry, we used the biotin/streptavidin method [15].

The following antibodies, assessed on 2-3 *μ*m thick sections cut from paraffin-embedded tissue (paraffin blocks) and put on special slides (Super frost, commercially available), were used: MUC 1 (Polyconal Antibody, Thermo Fisher Scientific), Ki 67 (Clone SP-6, Lab Vision, Thermo Fisher Scientific), CD 34 (Clone QBEnd/10, Lab Vision, Thermo Fisher Scientific), Factor VIII (Clone F8/86, Lab Vision, Thermo Fisher Scientific), CD 68 (Clone KP1, Lab Vision, Thermo Fisher Scientific), MMP-7 (Clone ID2, Lab Vision, Thermo Fisher Scientific), CEA (Clone COL-1, Lab Vision, Thermo Fisher Scientific), p21 (Clone CP74, Lab Vision, Thermo Fisher Scientific), and p53 (Clone DO-1, Lab Vision, Thermo Fisher Scientific). Evaluation of the immunohistochemical stains was independently performed by two pathologists.

To estimate the immunohistochemical results, the following scale was established:

- 0=negative staining (0-5% of the cells are stained)
- 1+=weak positivity staining (5-10% of the cells are stained)
- 2+=moderate positivity staining (10-50% of the cells are stained)
- 3+=strong positivity staining (over 50% of the cells are stained)

The percentage of positive cells was established by analyzing 500-1000 cells, and the ratio between the numbers of positive cells versus the total number of analyzed cells was represented. Immunohistochemical analysis of tumor's blood supply was made by using antibodies for CD 34 or Factor VIII. In order to the count positive markers for microvascularization, using literature criteria [16], it was considered not necessary for a vascular lumen to be present. There were 3 areas quantified at a magnitude of 100 (10x eyepiece, 10x objective) that were identified with the highest microvascularization, and the vessels were quantified at a magnitude of 200 (10x eyepiece, 20x objective).

### 2.3. Statistical Analysis

Univariate comparisons were performed using Pearson's chi-squared test and Student's *t*-test, as appropriate. All statistical tests were two sided, and a level of *p* < 0.05 was used to indicate statistical significance. Statistical analysis was performed using the Stata MP Version 13.0 (College Station, TX).

## 3. Results

The gender distribution for the patients included in this study is as follows: 133 males (57.8%) and 97 females (42.2%).

**Figure 1:**
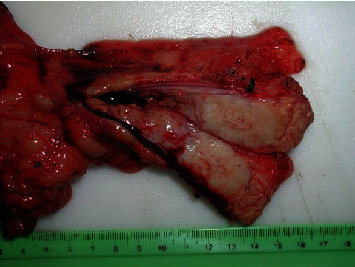
Surgically resected specimen: pancreatic tumor (gross appearance).

**Figure 2:**
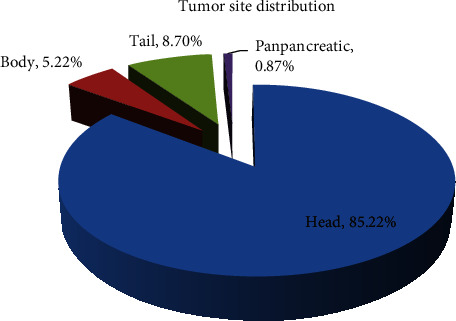
Tumor site distribution.

Tumor site distribution is as follows: in the pancreatic head, *n* = 196 (85.2%); in the pancreatic body, *n* = 12 (5.2%); in the pancreatic tail, *n* = 20 (8.7%); and for panpancreatic tumors, *n* = 2 (0.9%) (Figures 1 and 2).

Age distribution showed one (0.43%) case in the 20–29-year-old group, 12 (5.22%) cases in the 30–39-year-old group, 37 (16.08%) cases in the 40–49-year-old group, 77 (33.48%) cases in the 50–59-year-old group, 72 (31.32%) cases in the 60–69-year-old group, 30 (13.04%) cases in the 70–79-year-old group, and one (0.43%) case in the last group over 80 years old.

**Figure 3:**
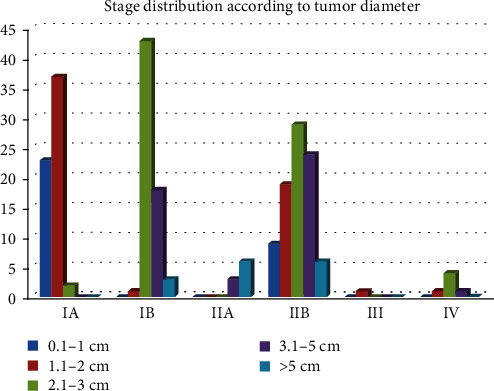
Stage distribution according to tumor diameter.

Tumor size distribution is as follows: 32 cases in the 0.1–1.0 cm group, 50 cases in the 1.1–2.0 cm group, 70 cases in the 2.1–3.0 cm, 46 cases in the 3.1–5.0 cm, and 15 cases in the last group of over 5 cm. In 17 cases, the size of the tumor could not be assessed (Figure 3).

According to the TNM staging system [1, 12, 13], tumor stage distribution was as follows: 38.3% in the pT1 group, 55.2% in the pT2 group, 6.1% in the pT3 group, and 0.4% in the pT4 group; 27.0% of the cases were stage IA, 28.3% were stage IB, 3.9% were stage IIA, 37.8% were stage IIB, 0.4% were stage III, and 2.6% were stage IV.

Correlation between pTNM stage and tumor size can be seen in Figure 3.

According to grade distribution, G1 was encountered in 56% of the cases, G2 was encountered in 29%, and G3 was encountered in 15%.

**Figure 4:**
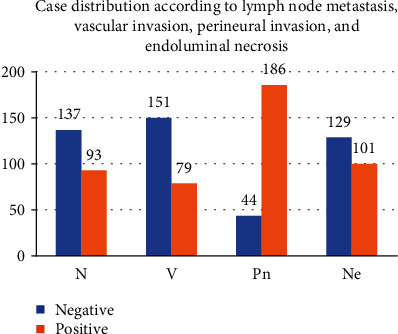
Distribution of lymph node metastasis (N), vascular invasion (V), perineural invasion (Pn), and endoluminal necrosis (Ne).

**Figure 5:**
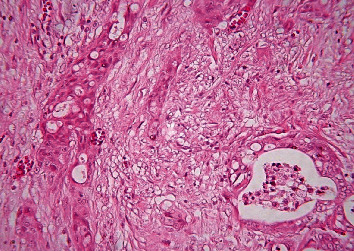
Pancreatic ductal adenocarcinoma, endoluminal necrosis, hematoxylin-eosin staining, ×200.

The other histopathological features observed, namely, the presence/absence of lymph node metastasis (N0/N1), the presence/absence of vascular invasion (V+/V-), the presence/absence of perineural invasion (Pn+/Pn-), and the presence/absence of necrosis in the tumoral glandular lumen (Ne+/Ne), are represented in Figures 4 and 5.

Fifteen out of 230 cases of pancreatic ductal carcinoma were diagnosed as histological variants represented by adenosquamous carcinoma (*n* = 3), mucinous carcinoma (*n* = 5), undifferentiated carcinoma with osteoclast-like giant cells (*n* = 3), and undifferentiated (anaplastic) carcinoma with fusiform cells (*n* = 4).

**Figure 6:**
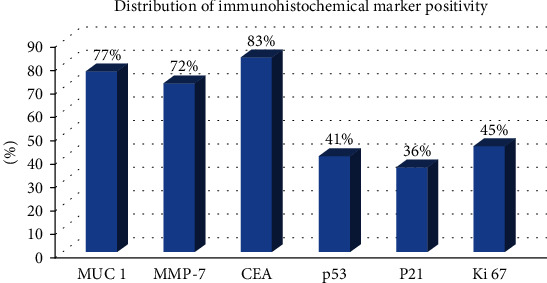
Distribution of immunohistochemical marker positivity.

The positivity of the immunohistochemical markers used is represented in Figure 6 and Table 1.

**Figure 7:**
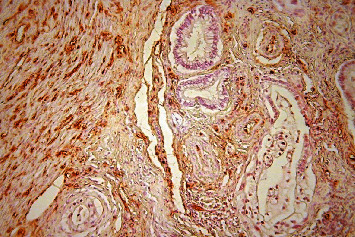
Pancreatic ductal adenocarcinoma, hypervascular area, immunohistochemical staining, Factor VIII, ×200.

**Figure 8:**
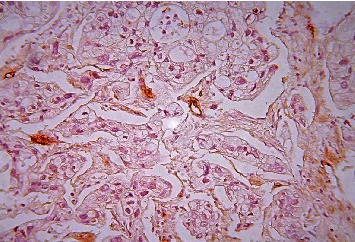
Pancreatic ductal adenocarcinoma, hypovascular area, immunohistochemical staining, Factor VIII, ×200.

**Figure 9:**
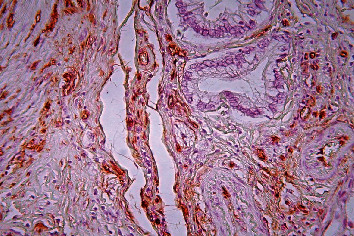
Pancreatic ductal adenocarcinoma, hypervascular area, immunohistochemical staining, CD 34, ×200.

**Figure 10:**
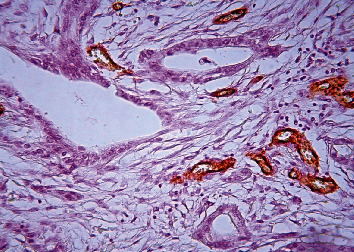
Pancreatic ductal carcinoma, hypovascular area, immunohistochemical staining, CD 34, ×200.

The microvascular density was appreciated using CD 34 and Factor VIII markers (Figures 7–10). For each case, the same number of serial sections were used, and the maximum distribution of microvascular density, with the cutoff value being 30.1 according literature data [15], was established. All studied features were analyzed depending on two categories: hypervascularity and hypovascularity (Table 2). A higher distribution of vessels around the tumor glands was observed (Figures 7 and 9).

*p* < 0.05. s.s.: statistically significant; s.i.: statistically insignificant. ^a^Method used: Student *t*-test. ^b^Method used: Pearson's chi-squared test.

A statistically significant correlation between microvascular density and age, gender, tumor site or size, and presence of tumor metastases could not be established. However, a higher microvascularization in advanced stages was observed (Table 2). For both intratumoral and peritumoral inflammatory infiltrates, we have used an immunohistochemical technique using the antibody for CD 68, which is expressed on macrophages and monocytes.

After data analysis, some of the following correlations could be established (Table 3):

1. Patients with early stages (IA and IB), with low-grade malignancy (G1), and with small tumor size (1-1.5 cm) localized in the head of pancreas; p53—negative; p21—positive; CD 68—positive in peritumoral tissue; Ki 67 < 5%; without metastases at the time of surgery—had a better prognosis with a survival rate of about 7 months. This is why there is a need to focus on improving risk stratification and identifying early stage disease or premalignant lesions, while they are still resectable [7]
2. Patients with tumors bigger than 5 cm, localized in the body or/and tail; patients with tumors bigger than 5-6 cm, even localized in the head; p21—negative; p53—positive; Ki 67 in 30-35% of tumoral cells; with endoluminal necrosis, intravascular tumoral emboli, and presence of metastases—had a poor prognosis and increased risk for metastases (when these are absent at the time of diagnosis) and recurrences
3. Presence of perineural invasion and number of affected regional lymph nodes could not be correlated with prognostic factors or survival [17]
4. *CD 68* was positive in peritumoral tissue, in about 55-60% of well-differentiated G1 cases. The interaction between pancreatic cancer cells and tumor-associated macrophages remains to be established and may play a pivotal role in the progression of pancreatic cancer [18, 19]

The immunohistochemical panel is useful for an accurate diagnosis, staging, differential diagnosis, and establishment of additional prognostic factors that might be important.

The existence of a viable, stable, and specific marker is mandatory for the detection early disease, for preventing recurrences, for prolonging lifetime, and for formulating an adequate therapy.

## 4. Discussion

Pancreatic ductal adenocarcinoma (PDAC) is the most frequent pancreatic neoplasm and represents 80-90% of all malignant pancreatic tumors [1]. Incidence is about 50% higher in men than in women and in patients with 60-80 years of age [1]. In our cases, a slight male predominance has been observed in patients with 50-70 years of age.

According to the literature, the majority of ductal adenocarcinomas are located in the head of pancreas (60-70%); in our study, the majority of cases (85.2%) had the same location [2–4, 8].

An important tool in the evaluation of prognosis and guidance of a treatment is pTNM staging [13, 14].

The establishment of additional histopathological parameters, besides the usual ones, included in the used classifications, such as the presence of an intravascular tumoral emboli, perineural invasion, and endoluminal necrosis, are useful for the accuracy of the diagnosis [17, 20]. The presence of numerous intravascular tumoral emboli has to be considered as a poor prognostic factor [21]. In our study, tumoral emboli were present in 59 (34.3%) cases. The presence of lymph node metastases is also an important prognostic factor, without known correlation with the number of affected lymph nodes [16, 22]. In our study, 93(40.4%) cases had lymph node metastasis. Progression of tumoral stage represents a poor prognostic factor.

We found an increase in microvascularization in advanced stages, and studies have shown that angiogenic markers such as serum levels of VEGF and bFGF correlated with the stage, tumor diameter, and proliferation markers (Ki 67). These markers may also reflect disease progression [23, 24].

Most of the studied ductal adenocarcinomas were well differentiated, less than 2 cm, located in the head of the pancreas, without lymph node metastases, and had a better prognosis and a longer survival rate (more than 7 months) Table 3.

**Figure 11:**
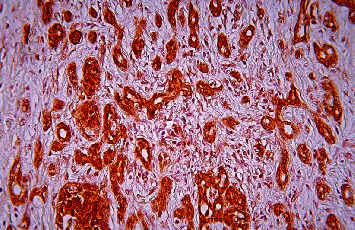
Immunohistochemical staining, MMP-7 (3+), ×100.

**Figure 12:**
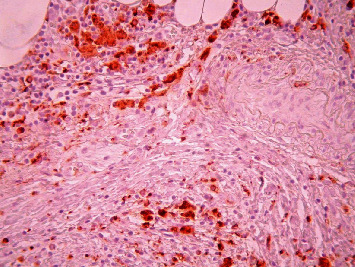
Pancreatic ductal carcinoma, inflammatory infiltrate, intratumoral and peritumoral (predominant) tissues, immunohistochemical staining, CD 68, ×200.

Immunohistochemistry with an increased number of antibodies is very useful; in studied cases, there were antibodies included that underline the following issues:

1. *MUC 1* was positive in 77% of the cases, with a staining index between 35 and 85% (2+ and 3+ on the staining scale) (Table 1), frequently in well-differentiated cases, independent of the number of lymph node metastases or disease stage. It is expressed usually in the luminal membrane (e.g., in the duct forming areas) and in the cytoplasm (e.g., in poorly differentiated areas); thus, it is considered a marker of aggressiveness [11].
2. *MMP-7* was positive in 72% of the cases, with a staining index between 25 and 75% (2+ and 3+ on the staining scale) (Figure 11); it was more frequent in well-differentiated cases, in early stages without lymph node metastases.
3. *CEA* was positive in 83% of the cases, with a staining index between 45 and 85% (2+ and 3+ on the staining scale) (Table 1).
4. *p53* was positive in 41% of the cases (staining index: 1+ and 2+), 25-30% in stages IA and IB, 45% in stages IIB and III, and 30-33% in stage IV; it was negative in adenosquamous carcinoma and mucinous carcinoma (PDC variants). In cases with a 3–5-month survival rate, it was frequently positive; in majority of the cases with a longer survival rate (more than 8 months), it was negative.
5. *P21* was positive in 36% of the cases, more frequent in early stages and in patients with low-grade carcinoma, G1. P21 is the downstream target of p53 activation and enables the DNA repair mechanisms by inducing G1 arrest. Despite the loss of its activity, its direct role in survival has not been proven yet [25].
6. *CD 68* was positive in peritumoral tissue, in about 55-60% of the well-differentiated G1 cases (Figure 12).
7. *Ki 67* was positive in about 40–45% of the cases with high-grade carcinoma (G2, G3) with short survival rate, and in cases with the presence of metastases at the time of surgical resection. There was a correlation between the number of positive cells and tumor's grade and stage.

A survival rate could be established in only 134 patients (due to lost follow-up, poor communication with oncologists, and lack of response/cooperation from patient's relatives), with an average survival rate of 12.96 months, without a good correlation with studied factors.

In studied cases, PanIN lesions were not as frequently encountered (47%) as they were presented in the literature, where they might be encountered in until 82% of cases [26–32]. An explanation for this could be the advanced stage of disease at the time of surgery [31].

## 5. Conclusions

It is recommended to study peritumoral/junction tissue from quantitative and qualitative points of view, to increase the number of prognostic factors.

This study represents a multidisciplinary approach, and it is a result of teamwork. It presents histopathological methods of examination of this severe illness and describes only a part of the scientific effort to determine main pathological mechanisms of this neoplasm.

## Figures and Tables

**Table 1:** Staining index, according to the scale used (0-3+) for the first 3 markers with the highest positivity.

	MUC 1	MMP-7	CEA
0	0-10%	0-10%	0-15%
1+	10-35%	10-25%	15-45%
2+	35-60%	25-50%	45-60%
3+	60-85%	50-75%	60-85%

**Table 2:** Microvascular analysis according to number of cases and clinicopathological features.

	No. of cases (*n* = 230)	Microvascularity		*p* value^∗^
		Hypervascularity (*n* = 108)	Hypovascularity (*n* = 122)	
Average age (years)	Mean ± SD	57.4 ± 7.9	59.0 ± 8.3	s.i.^a^
Gender				
F	97 (42.2%)	44	53	s.i^b^
M	133 (57.8%)	64	69	s.i.^b^
Site				
Head	196 (85.2)	94	102	s.i.^b^.
Body	12 (5.2%)	5	7	s.i.^b^
Tail	20 (8.7%)	8	12	s.i.^b^
Panpancreatic	2 (0.9%)	1	1	s.i.^b^
Liver metastasis				
Yes	6 (2.6%)	4	2	s.i.^b^
No	224 (97.4%)	104	120	
pT (AJCC)				s.i.^b^
T1	88 (38.3%)	42	46	
T2	127 (55.2%)	59	68	
T3	14 (6.1%)	6	8	
T4	1 (0.4%)	1	0	
Stage				*p* = 0.005 s.s^b^
IA	62 (27%)	24	38	
IB	65 (28.3%)	22	43	
IIA	9 (3.9%)	4	5	
IIB	87 (37.8%)	53	34	
III	1 (0.4%)	1	0	
IV	6 (2.6%)	4	2	

**Table 3:** Prognostic factors according to clinically, histopathologically, and immunohistochemically studied parameters (EN: endoluminal necrosis; ITE: intravascular tumoral emboli).

Prognosis	Stages	Tumor grade	Tumor size (cm)	Tumor localization	p53	p21	CD 68	Ki 67	Metastasis	EN, ITE
Better	IA; IB	G1	1-1.5	Head of pancreas	—	+	Present in peritumoral tissue	<5%	No	No
Poor			>5	Body or/and tail	+	—	Absent in peritumoral tissue	>30-35%	Yes	Yes
		G2, G3	>5-6	Body or/and tail or head						

## Data Availability

All the data to support the findings of this study are available from the corresponding author upon request.
